# Interobserver variability in preclinical assessment of collision variables following traffic accidents

**DOI:** 10.1007/s00068-024-02528-5

**Published:** 2024-04-13

**Authors:** Michael Hetz, Julius Rosch, Thomas Unger, Manuel F. Struck, Klaus-Dieter Schaser, Christian Kleber

**Affiliations:** 1 Department of Operative Medicine (DOPM), Clinic and Polyclinic for Orthopedics, Trauma Surgery and Plastic Surgery, University Hospital Leipzig, Liebigstr. 20, 04103 Leipzig, Germany; 2 Department of Orthopedics and Trauma Surgery, St Josef Hospital Essen-Werden, University Hospital Essen, Essen, Germany; 3 Verkehrsunfallforschung an der TU Dresden GmbH, Dresden, Germany; 4 Department of Anesthesiology and Intensive Care Medicine, University Hospital Leipzig, Leipzig, Germany; 5 University Center for Orthopedics, Traumatology, and Plastic Surgery, Carl Gustav Carus University Hospital Dresden, Dresden, Germany

**Keywords:** Assessment, Collision variables, Interobserver variability, Traffic accident, Injury prediction, eCall

## Abstract

**Purpose:**

Traffic accidents persist as a leading cause of death. European law mandates the integration of automatic emergency call systems (eCall). Our project focuses on an automated injury prediction device for car accidents, correlating technical and epidemiological input data, such as age, gender, seating position, impact on the passenger compartment, seatbelt usage, impact direction, EES, vehicle class, and airbag deployment. This study aims to explore interobserver variability in data collection quality in real accident scenarios. The assessment will evaluate the impact of user training and measure the time needed for data collection to inform user recommendations for the prehospital assessment.

Insights from this study can aid in evaluating the ability of different professional groups to identify potential accident-independent parameters at accident scenes. This includes, among other things, relaying information to dispatchers at rescue control centers, also within the context of telemedicine approaches.

**Methods:**

During group sessions, real accident scenarios were presented both before and after a training presentation. Participants, including laypersons, accident research staff, emergency services, hospital physicians, and emergency physicians, visually assessed injury prediction parameters within a time limit. Training involved defining and explaining parameters using accident images. The study analyzed participant demographics, prediction accuracy, and time required, comparing assessment quality between professional groups and before and after training.

**Results:**

In summary, the study demonstrates that training had a significantly positive impact on the quality of assessment for technical accident parameters. The processing time decreased significantly after training. A notable training effect was observed, particularly for the parameters of rigid collision object, affected passenger compartment, energy equivalent speed (EES), and front and side airbags. It was recommended that individuals without prior knowledge should receive training on assessing EES. Overall, it was evident that technical parameters following a traffic accident can be well assessed through training, irrespective of the professional group.

**Conclusion:**

Significant differences in the assessment quality of technical accident parameters were observed based on technical and medical expertise. After user training, interdisciplinary differences were reconciled, and all professional groups yielded comparable results, indicating that training can enhance the assessment abilities of all participants in the rescue chain, while the time required for assessing accident parameters was significantly reduced with training.

## Introduction

Despite improvements in vehicle technology, emergency services, and trauma management, traffic accidents remain one of the leading causes of death, with a shifting mortality trend towards the prehospital phase [1]. According to European law, since 2018, every new passenger car model must be equipped with automatic emergency call systems (eCall) [2]. Currently, eCall transmits neither medical nor occupant-specific information to emergency call centers.

Our project team is developing an automated injury prediction device for car accidents and therefore an injury prediction tool for traffic accidents in another project. After entering clinical, quickly assessable accident parameters such as seating position, impact against the passenger cell, seatbelt usage, impact direction, EES (energy equivalent speed), vehicle class, and airbag deployment, an expected injury pattern of the vehicle occupants is generated based on an injury risk function derived from logistic regression (unpublished results from our research group). Although the data required for injury prediction are technically measurable, they are currently not comprehensively collected or stored by vehicles or rescue teams. For these reasons, it is necessary for the input data to be initially captured at the accident scene by the attending personnel. This is currently being done in a prospective, multicenter offline testing of the injury prediction tool.

The aim of this subproject is to investigate the ability to collect traffic accident–specific data at the accident scene and reveal potential interobserver variability in data collection quality among different professional groups using real accident scenarios. Furthermore, the time required to conduct data collection will be assessed. Based on these findings, user recommendations for the offline tool will be formulated.

Insights from this study can help evaluate the ability of different professional groups to identify potential accident-independent parameters at accident scenes. This could include, among other things, relaying information to dispatchers at rescue control centers, also within the context of telemedicine approaches. Additionally, the time required and the learning effects from training sessions could provide guidance for possible user training in such scenarios.

## Materials and methods

This study is a prospective descriptive cohort study. It has received a favorable vote from the ethics committee (BO-EK-207042021).

### Participants

A total of 50 participants were surveyed, divided into five groups with ten individuals each. The positive control group consisted of employees of the Traffic Accident Research Institute at the Technical University of Dresden (hereinafter referred to as TAR, Traffic Accident Researcher). These individuals have professional experience in technical accident reconstruction, are familiar with the variables to be collected, and regularly review images of real accident scenarios. The negative control group comprised ten laypersons with no experience in emergency medicine or the technical assessment of accident scenarios (LAY). Other professional groups included emergency physicians (EP), hospital physicians from a university-level trauma center (HP), and emergency services (EC; each with *n* = 10). The survey was conducted pseudonymously, and data on the participants’ age, gender, professional group, and years of professional experience were collected.

### Survey and training

**Fig. 1:**
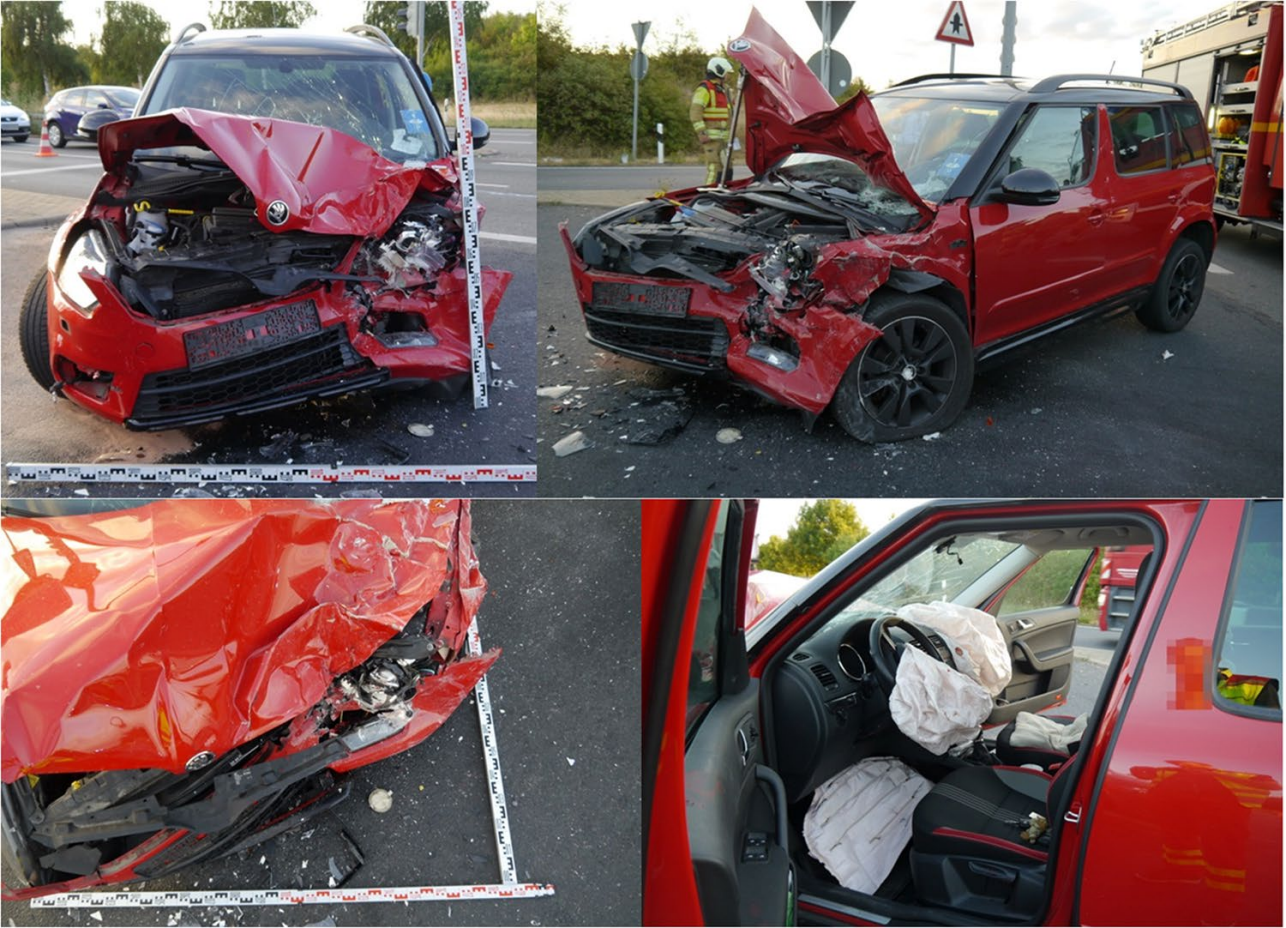
Exemplary images from the participant survey. Depicted is a crash vehicle, showing the damage pattern, seating position, and airbag status

During the participant survey, group sessions of approximately ten individuals per survey session were conducted. Real accident scenario images were presented both before and after a training presentation using PowerPoint as exemplified in (Fig. 1) (Microsoft Corporation, Washington, USA). Participants were asked to visually assess the required input parameters for injury prediction (similar to a real accident scenario) and were given a time limit of 120 s per case. The following accident parameters had to be evaluated, as they are currently necessary for injury prediction: vehicle class (compact, mid-size, luxury), whether the collision involved a rigid obstacle (e.g., tree, wall), rollover, main deformation area of the vehicle to determine the impact direction (front, right, left, rear), impact on the passenger compartment (impact between A and B pillars), energy equivalent speed (EES), seating position of the affected person (driver or passenger), seatbelt usage, age (categorized as 0–17 years, 18–64 years, > 65 years), gender, and airbag deployment (differentiating between curtain, frontal, knee, and side airbags). Additionally, two complex accident scenarios (rollover, multiple collisions) were demonstrated before and after the training.

During the training session, which took place after the first ten cases, the following parameters were defined and explained using real accident images in a presentation: vehicle class, rigid obstacle, rollover, impact on the passenger compartment, impact point (front, right, left, rear), seatbelt status, four airbags (curtain airbag, frontal airbag, knee airbag, side airbag), and energy equivalent speed (EES). Participants had the opportunity to ask questions during the training.

The parameters (driver/passenger), age, gender, and seatbelt status were provided to participants both before and after the training, as they could not be determined from the accident images. Estimated values were recorded on questionnaires. The processing time for all 24 cases was recorded by 23 randomly selected participants (2 laypersons, 3 traffic accident research staff, 5 emergency medical service personnel, 7 hospital doctors, 6 emergency physicians).

Participants were informed about the purpose and procedure of the survey and provided written consent.

### Scenarios

The scenarios used are part of the GIDAS database (German In-Depth Accident Study) collected by the Traffic Accident Research Institute at TU Dresden, and actual values of the variables to be collected are available to the study team as a reference. Fully reconstructed scenarios were utilized (*n* = 10 per professional group).

Out of the ten cases to be assessed, six had an EES of 0–30 km/h, three had an EES of 30–50 km/h, and one case had an EES of > 50 km/h. The selection of cases was done randomly and based on the relative distribution of EES in the GIDAS dataset (extraction 2010–2021). In this dataset, for maximum AIS 3 or more severe injuries (AIS3 +) following a traffic accident, the EES distributions were as follows: EES 0–30 (*n* = 1.309, 57%), EES 30–50 km/h (*n* = 609, 27%), EES > 50 km/h (*n* = 369, 16%). The relative distribution of impact direction from GIDAS (52% frontal, 27% side impact, ratio approximately 2:1) was also considered. Seven cases with frontal impact and three cases with side impact were selected for each group. Additionally, two complex cases (multiple collisions, rollover) were demonstrated before and after the training.

### Statistics

In addition to the descriptive analysis of data regarding the demographics of the respondents, prediction accuracy, and time required, the quality of individual variable assessments was compared between professional groups. Furthermore, a comparison was made between the quality of assessments before and after the training. Except for the EES value, the response options for the other 13 variables were considered binary (true vs. false response). For the EES estimation, the deviation from the actual reconstructed numerical value was considered, and the correctness of the EES estimation was assessed with a tolerance of 10 km/h. If the response fell within this range, it was considered correct. The Wilcoxon test was used for non-normally distributed, paired samples to calculate significances in differences between professional groups before and after the training. The Kruskal–Wallis test was applied to calculate significances in differences between the assessed parameters before and after the training. If significant differences were observed, professional group-specific differences were calculated using the Mann–Whitney *U* test. Differences were recognized as statistically significant at a significance level of *p* < 0.05. Additionally, a comparison was made between front and side impact. No imputation methods were applied for missing data. Excel for Mac (v16.46, Microsoft Corporation, Washington, USA), GraphPad Prism version 9.0.1 for Mac (GraphPad Software Inc., San Diego, CA, USA), and SPSS (release 23 for Windows, IBM, Armonk, NY, USA) were used to calculate and visualize the results.

## Results

A total of 50 participants were surveyed. Demographic data were available for 47 participants (94%). Complete data were available for the LAY, TAR, and EC groups (Table 1). In the HP group, data were missing for two participants and for one participant in the EP group. Of all study participants, 81% (*n* = 38) were male, and 19% (*n* = 9) were female. The mean age of all participants was 34 years (SD 9.08). The lowest mean age was 25 years in the LAY group, and the highest mean age was 43 years in the EP group. The mean professional experience was 8 years, with employees of TAR (12 years), EC (11 years), and EP (14 years) having a significantly longer average professional experience compared to HP (3 years).

**Table 1:** Demographic parameters of the surveyed participants (gender, age, and professional experience)

	*n*	Gender		Age (in years)		Professional experience (in years)			
		m	fm	M	SD	M	SD	Min	Max
LAY	10	10	0	25	1	-	-	-	-
TAR	10	10	0	39	7	12	3	5	15
EC	10	6	4	33	8	11	8	4	30
HP	8	4	4	30	3	3	3	0.5	6
EP	9	7	2	43	10	14	10	3	27
Total	47	37	10	34	9	8	8	0.5	30

*m* male, *fm* female, *M* mean, *SD* standard deviation

*LAY* laypersons, *TAR* Traffic Accident Research staff, *EP* emergency physicians, *HP* hospital physicians, *EC* emergency services

### Processing time per case

The average processing time per case was 68 s, with case 1 taking 96 s (ranging from 57 to 140 s) and the last processed case, case 22, taking 60 s (ranging from 37 to 98 s). The median processing time for the 10 non-complex cases before training (cases 1–10) was 73 s, and after training (cases 13–22), it was 59 s (*p* = 0.003, two-way ANOVA).

**Fig. 2:**
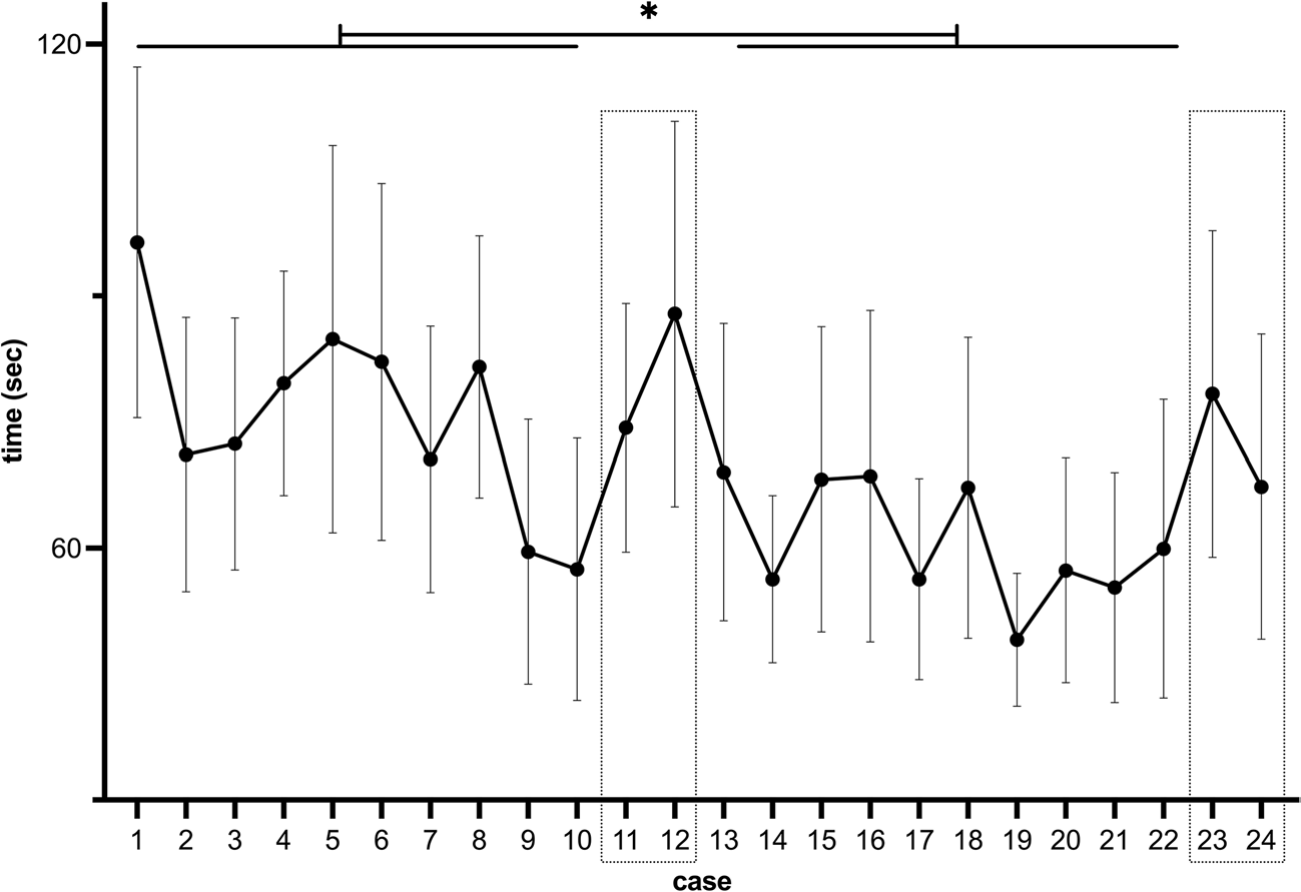
Processing time per case (mean and standard deviation). A trend of reduced processing time with case progression is evident, with the complex cases 11, 12, and 23, 24 (boxes) standing out as outliers. Cases before training (1–10) were significantly processed more slowly than after training (13–22, *p = 0.003, two-way ANOVA)

Figure 2 shows the decreasing trend in processing time with the number of cases performed. The complex cases required longer processing times. Cases 11 and 12 (before training) were completed on average after 81 s (ranging from 49 to 119 s). Cases 23 and 24 (after training) took an average of 72 s (ranging from 38 to 118 s).

### Impact of user training

Before training, of all participants, regardless of professional group and impact direction, 629 of the binary answers (10.7%, median 37 per parameter) were answered incorrectly. After training, this reduced to 340 answers (5.5%, median 11 per parameter). The sum of the differences between estimated and actual EES before training was 4250 km/h and decreased by 28.8% to 1223 km/h after training.

Regarding the quality of the input parameter assessments before vs. after training, regardless of impact direction, the following relative distributions were observed: vehicle class, 74% correct answers before and 74% after training; rigid obstacle collision, 90% before and 99% after training; rollover, 98% before and 98% after training; impact side, 91% before and 93% after training; damage to the passenger compartment, 71% before and 95% after training; EES within ± 10 km/h, 52% before and 74% after training; seatbelt usage, 97% before and 98% after training; curtain airbag, 88% before and 93% after training; front airbag, 96% before and 99% after training; knee airbag, 93% before and 94% after training; and side airbag, 80% before and 91% after training. Seat position, age group, seatbelt usage, and gender were predetermined for the participants (Table 2).

**Table 2:** Correct assessments by professional group, input parameters before and after user training, and the difference, with a breakdown by impact direction (relative distribution in %)

		Before training (in %)			After training (in %)			Delta (in %)	Wilcoxon test	
		Frontal	Side	Total	Frontal	Side	Total		*Z*	*p*
Vehicle class	LAY	67.1	86.7	73.0	64.3	73.3	67.0	− 6.0	− 0.933	0.351
	TAR	80.0	93.3	84.0	77.1	93.3	82.0	− 2.0	− 0.312	0.755
	EC	62.9	76.7	67.0	78.6	76.7	78.0	11.0	− 1.371	0.170
	HP	65.7	80.0	70.0	57.1	93.3	68.0	− 2.0	− 0.424	0.672
	EP	71.4	83.3	75.0	71.4	80.0	74.0	1.0	0.000	1.000
	Total	69.4	84.0	73.8	69.7	83.3	73.8	0.0	− 0.057	0.954
Rigid object	LAY	94.3	93.3	94.0	100.0	100.0	100.0	6.0	− 2.121	**0.034**
	TAR	98.6	90.0	96.0	100.0	100.0	100.0	4.0	− 1.633	0.102
	EC	97.1	83.3	93.0	98.6	96.7	98.0	5.0	− 1.890	0.059
	HP	76.7	76.7	76.0	97.2	100.0	98.0	22.0	− 2.539	**0.011**
	EP	92.9	93.3	93.0	97.1	100.0	98.0	5.0	− 1.518	0.129
	Total	91.7	87.3	90.4	98.6	99.3	98.8	8.4	− 4.278	** < 0.001**
Rollover	LAY	100.0	96.7	99.0	97.1	93.3	96.0	− 3.0	− 1.342	0.180
	TAR	100.0	96.7	99.0	98.6	90.0	96.0	− 3.0	− 0.816	0.414
	EC	100.0	93.3	98.0	100.0	100.0	100.0	2.0	− 1.000	0.317
	HP	94.3	93.3	94.0	100.0	96.7	99.0	5.0	− 1.633	0.102
	EP	100.0	96.7	99.0	100.0	93.3	98.0	− 1.0	− 1.000	0.317
	Total	98.9	95.3	97.8	99.1	94.7	97.8	0.0	− 1.82	0.856
Impact side	LAY	88.6	83.3	87.0	100.0	76.7	93.0	6.0	− 1.508	0.132
	TAR	95.7	96.7	96.0	98.6	93.3	97.0	1.0	− 0.447	0.655
	EC	90.0	96.7	92.0	100.0	73.3	92.0	0.0	0.000	1.000
	HP	84.3	90.0	86.0	94.3	76.7	89.0	3.0	− 1.134	0.257
	EP	91.4	93.3	92.0	98.6	76.7	92.0	0.0	0.0000	1.000
	Total	90.0	92.0	90.6	98.3	79.3	92.6	2.0	− 1.527	0.127
Compartment affected	LAY	68.6	60.0	66.0	97.1	90.0	95.0	29.0	− 2.840	**0.005**
	TAR	81.4	63.3	76.0	100.0	93.3	98.0	22.0	− 2.699	**0.007**
	EC	74.3	66.7	72.0	97.1	93.3	96.0	24.0	− 2.871	**0.004**
	HP	70.0	60.0	67.0	95.7	83.3	92.0	25.0	− 2.692	**0.007**
	EP	78.6	60.0	73.0	95.7	93.3	95.0	22.0	− 2.754	**0.006**
	Total	74.6	62.0	70.8	97.1	90.0	95.2	24.4	− 6.039	** < 0.001**
EES ± 10 km/h	LAY	45.7	76.7	55.0	61.4	83.3	68.0	13.0	− 1.897	0.058
	TAR	77.1	86.7	80.0	75.7	83.3	78.0	− 2.0	− 0.360	0.719
	EC	44.3	36.7	42.0	61.4	96.7	72.0	30.0	− 2.608	**0.009**
	HP	35.7	40.0	37.0	67.1	83.3	72.0	35.0	− 2.354	**0.019**
	EP	47.1	40.0	45.0	75.7	90.0	80.0	35.0	− 2.539	**0.011**
	Total	50.0	56.0	51.8	68.3	87.3	74.0	22.2	− 4.522	** < 0.001**
Seating position*	LAY	100.0	96.7	99.0	100.0	100.0	100.0	1.0	− 1.000	0.317
	TAR	100.0	100.0	100.0	100.0	100.0	100.0	0.0	0.000	1.000
	EC	98.6	93.3	97.0	98.6	96.7	98.0	1.0	− 1.000	0.317
	HP	98.6	100.0	99.0	100.0	100.0	100.0	1.0	− 1.000	0.317
	EP	100.0	96.7	99.0	100.0	100.0	100.0	1.0	− 1.000	0.317
	Total	99.4	97.3	98.8	99.7	99.3	99.6	0.8	− 2.000	0.046
Seatbelt usage*	LAY	98.6	90.0	96.0	95.7	100.0	97.0	1.0	− 0.276	0.783
	TAR	98.6	100.0	99.0	97.1	100.0	98.0	− 1.0	− 0.577	0.564
	EC	91.4	93.3	92.0	95.7	100.0	97.0	5.0	− 1.667	0.096
	HP	95.7	100.0	97.0	100.0	100.0	100.0	3.0	− 1.732	0.083
	EP	100.0	100.0	100.0	98.6	100.0	99.0	− 1.0	− 1.000	0.317
	Total	96.9	96.7	96.8	97.4	100.0	98.2	1.4	− 1.334	0.182
Age*	LAY	100.0	100.0	100.0	100.0	100.0	100.0	0.0	0.000	1.000
	TAR	100.0	100.0	100.0	100.0	100.0	100.0	0.0	0.000	1.000
	EC	97.1	100.0	98.0	100.0	100.0	100.0	2.0	− 1.000	0.317
	HP	100.0	100.0	100.0	100.0	100.0	100.0	0.0	0.000	1.000
	EP	98.6	100.0	99.0	100.0	100.0	100.0	1.0	− 1.000	0.317
	Total	99.1	100.0	99.4	100.0	100.0	100.0	0.6	− 1.342	0.180
Gender*	LAY	100.0	100.0	100.0	100.0	100.0	100.0	0.0	− 1.000	0.317
	TAR	100.0	100.0	100.0	100.0	100.0	100.0	0.0	− 0.577	0.564
	EC	98.6	100.0	98.0	100.0	100.0	100.0	2.0	− 1.000	0.317
	HP	98.6	100.0	99.0	100.0	100.0	100.0	1.0	− 1.000	0.317
	EP	98.6	100.0	99.0	100.0	100.0	100.0	1.0	− 0.577	0.564
	Total	99.1	100.0	99.0	100.0	100.0	100.0	1.0	1.000	0.999
Curtain airbag	LAY	81.4	76.7	80.0	91.4	83.3	89.0	9.0	− 0.718	0.473
	TAR	98.6	90.0	96.0	98.6	96.7	98.0	2.0	− 0.557	0.577
	EC	94.3	76.7	89.0	94.3	90.0	93.0	4.0	− 0.862	0.389
	HP	90.0	80.0	87.0	91.4	90.0	91.0	4.0	− 0.073	0.942
	EP	92.9	80.0	89.0	92.9	96.7	94.0	5.0	− 1.508	0.132
	Total	91.4	80.7	88.2	93.7	91.3	93.0	4.8	− 1.387	0.166
Front airbag	LAY	95.7	73.3	89.0	98.6	93.3	97.0	8.0	− 2.333	**0.020**
	TAR	100.0	100.0	100.0	100.0	100.0	100.0	0.0	0.000	1.000
	EC	98.6	93.3	97.0	100.0	100.0	100.0	3.0	− 1.000	0.317
	HP	97.1	90.0	95.0	100.0	100.0	100.0	5.0	− 1.890	0.059
	EP	100.0	96.7	99.0	100.0	100.0	100.0	1.0	− 1.000	0.317
	Total	98.3	90.7	96.0	99.7	98.7	99.4	3.4	− 3.213	**0.001**
Knee airbag	LAY	81.4	80.0	81.0	88.6	96.7	91.0	10.0	− 0.679	0.497
	TAR	100.0	100.0	100.0	94.3	100.0	96.0	− 4.0	− 2.000	**0.046**
	EC	94.3	96.7	95.0	95.7	100.0	97.0	2.0	− 0.632	0.527
	HP	91.4	90.0	91.0	87.1	100.0	91.0	0.0	− 0.632	0.527
	EP	94.3	100.0	96.0	91.4	100.0	94.0	− 2.0	− 0.816	0.414
	Total	92.3	93.3	92.6	91.4	99.3	93.8	1.2	− 0.603	0.547
Side airbag	LAY	82.9	43.3	71.0	95.7	70.0	88.0	17.0	− 1.980	**0.048**
	TAR	95.7	76.7	90.0	100.0	90.0	97.0	7.0	− 1.933	0.053
	EC	92.9	36.7	76.0	91.4	66.7	84.0	8.0	− 1.381	0.167
	HP	94.3	43.3	79.0	95.7	76.7	90.0	11.0	− 2.598	**0.009**
	EP	97.1	53.3	84.0	100.0	80.0	94.0	10.0	− 2.058	**0.040**
	Total	92.6	50.7	80.0	96.6	76.7	90.6	10.6	− 4.196	** < 0.001**

Comparison regardless of impact direction using the Wilcoxon test (bold indicates *p* < 0.05)

*Predetermined values

*LAY* laypersons, *TAR* Traffic Accident Research staff, *EP* emergency physicians, *HP* hospital physicians, *EC* emergency services

#### All impact directions

Regardless of the impact direction, after training, LAY (− 6%), TAR, and hospital doctors (− 2% each) showed slightly poorer assessments of the vehicle class (not significant, *p* > 0.315). Significant improvements were observed in the assessment of a rigid obstacle collision by LAY (+ 6%, *p* = 0.034) and hospital doctors (+ 22%, *p* = 0.01), with a significant overall improvement among all professional groups (+ 8.4%, *p* < 0.01). After training, all professional groups exhibited significantly better assessments of passenger compartment damage (+ 20%, *p* < 0.007). The estimation of EES was also significantly better for EC (+ 30%, *p* = 0.009), hospital doctors (+ 35%, *p* = 0.019), EP (+ 35%, *p* = 0.011), and in the overall assessment (+ 22%, *p* < 0.001). In terms of assessing airbag deployments, significantly better results were obtained for the assessment of front airbag deployment by LAY (+ 8%, *p* = 0.02), as well as the assessment of side airbag deployment by LAY (+ 17%, *p* = 0.048), hospital doctors (+ 11%, *p* = 0.09), and EP (+ 10%, *p* = 0.04).

#### Frontal impact

Looking at the cases after a frontal collision, in addition to the overall assessment mentioned earlier, significant differences were observed in the assessment of impact direction by LAY (*p* = 0.023), EC (*p* = 0.008), and hospital doctors (*p* = 0.02). Laypersons also showed a significantly better estimation of the EES value (*p* = 0.024). Compared to the overall assessment (Table 2), differences in front and side airbag assessments by LAY were not significant.

#### Side impact

For side impact, after training, EC showed a significantly better assessment of rigid obstacle collision (*p* = 0.046), impact direction (*p* = 0.02), and side airbag (*p* = 0.03). Hospital doctors (*p* = 0.046) and EP (*p* = 0.025) also had better scores for impact direction. Compared to the overall assessment (Table 2), LAY did not show significant differences in the assessment of rigid obstacle collision, front and side airbags. For TAR, knee airbag, and EP, side airbag assessments were not significant.

#### Complex cases

Compared to the overall assessment (Table 2), LAY showed significantly better results after training for impact direction (*p* = 0.023), EES (*p* = 0.025), seatbelt usage (*p* = 0.008), and curtain airbag (*p* = 0.034). The significance for front and side airbags was not observed. TAR did not show significant differences, but EC showed significant differences in impact direction (*p* = 0.014) and curtain, front, and knee airbags (*p* = 0.046), with EES not being significant. Hospital doctors showed significant differences in rollover (*p* = 0.038) and impact direction (*p* = 0.005), while passenger compartment, EES, and side airbag assessments were no longer significant. Emergency physicians performed significantly better in assessing impact direction (*p* = 0.024) and front airbag (*p* = 0.025), with differences in EES and side airbag no longer being significant.

### Interdisciplinary comparison before and after user training

The following analysis pertains to the 20 non-complex cases. When considering differences in the assessed input parameters before training, significant differences between professional groups are observed in the assessment of vehicle class (*p* = 0.047), rigid obstacle collision (*p* = 0.036), impact side (*p* = 0.032), EES (*p* = 0.006), seatbelt usage (*p* = 0.024), front airbag (*p* = 0.01), and side airbag (*p* = 0.017). Of these, only the assessment of front airbag remains as the sole significant difference after training (*p* = 0.014; Table 3).

**Table 3:** Comparison between input parameters before and after user training

	Before training		After training	
	*H*	*p*	*H*	*p*
Vehicle class	9.66	**0.047**	6.92	0.140
Rigid object	10.25	**0.036**	4.45	0.348
Rollover	3.00	0.558	5.65	0.227
Impact side	10.53	**0.032**	9.51	0.050
Compartment affected	4.90	0.297	3.10	0.541
EES ± 10 km/h	14.44	**0.006**	4.12	0.390
Seating position*	2.31	0.679	4.00	0.406
Seatbelt usage*	11.26	**0.024**	3.73	0.443
Age*	3.06	0.547	0.00	1.000
Gender*	1.07	0.899	4.36	0.360
Curtain airbag	3.95	0.413	7.43	0.115
Front airbag	13.33	**0.010**	12.51	**0.014**
Knee airbag	5.02	0.286	5.61	0.231
Side airbag	12.06	**0.017**	7.48	0.113

*Preset values, *H* = *H* statistic, Kruskal–Wallis test

*LAY* laypersons, *TAR* Traffic Accident Research staff, *EP* emergency physicians, *HP* hospital physicians, *EC* emergency services

#### Interdisciplinary differences before training

In the further differentiation of the significantly differing input parameters, the interdisciplinary differences before training are presented in Table 4. Regarding the assessment of the vehicle class, TAR significantly outperformed LAY (*p* = 0.023) and EC. For rigid obstacle collision, hospital doctors performed significantly worse than LAY (*p* = 0.023), TAR (*p* = 0.007), and EP (*p* = 0.043). Impact side was rated significantly better by TAR compared to hospital doctors (*p* = 0.011). The EES was also significantly better estimated by TAR than by LAY (*p* = 0.009), EC (*p* = 0.005), hospital doctors (*p* = 0.001), and EP (*p* = 0.011). Seatbelt usage was predetermined; however, EP outperformed EC (*p* = 0.023). Concerning the front airbag, LAY were inferior to TAR (*p* = 0.023) and EP (*p* = 0.043). Side airbag was significantly better assessed by TAR compared to LAY (*p* = 0.019), EC (*p* = 0.003), and hospital doctors (*p* = 0.019).

**Table 4:** Interdisciplinary differences before user training, subdivided by input parameters (which showed significances in the Kruskal–Wallis test, Mann–Whitney *U* test)

		*U*	*Z*	*p*
Vehicle class	**LAY vs. TAR**	20.50	− 2.31	**0.023**
	LAY vs. EC	39.00	− 0.86	0.436
	LAY vs. HP	46.00	− 0.31	0.796
	LAY vs. EP	46.50	− 0.27	0.796
	**TAR vs. EC**	10.50	− 3.09	**0.002**
	TAR vs. HP	25.50	− 1.94	0.063
	TAR vs. EP	30.00	− 1.56	0.143
	EC vs. HP	38.00	− 0.93	0.393
	EC vs. EP	36.50	− 1.05	0.315
	HP vs. EP	47.50	− 0.19	0.853
Rigid object	LAY vs. TAR	41.00	− 0.78	0.529
	LAY vs. EC	47.50	− 0.21	0.853
	**LAY vs. HP**	20.50	− 2.34	**0.023**
	LAY vs. EP	47.50	− 0.21	0.853
	TAR vs. EC	39.50	− 0.91	0.436
	**TAR vs. HP**	15.50	− 2.74	**0.007**
	TAR vs. EP	39.50	− 0.91	0.436
	EC vs. HP	23.50	− 2.08	0.043
	EC vs. EP	50.00	0.00	1.000
	**HP vs. EP**	23.50	− 2.08	**0.043**
Impact side	LAY vs. TAR	24.00	− 2.14	0.052
	LAY vs. EC	37.50	− 1.04	0.353
	LAY vs. HP	45.00	− 0.41	0.739
	LAY vs. EP	38.00	− 1.07	0.393
	TAR vs. EC	33.00	− 1.45	0.218
	**TAR vs. HP**	17.00	− 2.68	**0.011**
	TAR vs. EP	30.00	− 1.78	0.143
	EC vs. HP	30.00	− 1.67	0.143
	EC vs. EP	49.00	− 0.09	0.971
	HP vs. EP	29.00	− 1.87	0.123
EES ± 10 km/h	**LAY vs. TAR**	16.50	− 2.56	**0.009**
	LAY vs. EC	35.50	− 1.10	0.280
	LAY vs. HP	28.00	− 1.68	0.105
	LAY vs. EP	36.50	− 1.03	0.315
	**TAR vs. EC**	14.50	− 2.70	**0.005**
	**TAR vs. HP**	9.00	− 3.12	**0.001**
	**TAR vs. EP**	17.00	− 2.52	**0.011**
	EC vs. HP	44.50	− 0.42	0.684
	EC vs. EP	47.50	− 0.19	0.853
	HP vs. EP	40.00	− 0.77	0.481
Seatbelt usage*	LAY vs. TAR	39.50	− 1.14	0.436
	LAY vs. EC	35.00	− 1.26	0.280
	LAY vs. HP	48.50	− 0.14	0.912
	LAY vs. EP	35.00	− 1.83	0.280
	TAR vs. EC	24.00	− 2.33	0.052
	TAR vs. HP	40.00	− 1.09	0.481
	TAR vs. EP	45.00	− 1.00	0.739
	EC vs. HP	32.00	− 1.53	0.190
	**EC vs. EP**	20.00	− 2.81	**0.023**
	HP vs. EP	35.00	− 1.83	0.280
Front airbag	**LAY vs. TAR**	20.00	− 2.80	**0.023**
	LAY vs. EC	27.00	− 2.04	0.089
	LAY vs. HP	36.50	− 1.11	0.315
	**LAY vs. EP**	23.50	− 2.36	**0.043**
	TAR vs. EC	45.00	− 1.00	0.739
	TAR vs. HP	30.00	− 2.17	0.143
	TAR vs. EP	45.00	− 1.00	0.739
	EC vs. HP	37.00	− 1.29	0.353
	EC vs. EP	49.50	− 0.07	0.971
	HP vs. EP	34.50	− 1.55	0.247
Side airbag	**LAY vs. TAR**	19.50	− 2.39	**0.019**
	LAY vs. EC	49.00	− 0.08	0.971
	LAY vs. HP	44.00	− 0.48	0.684
	LAY vs. EP	35.00	− 1.19	0.280
	**TAR vs. EC**	12.50	− 2.96	**0.003**
	**TAR vs. HP**	19.00	− 2.50	**0.019**
	TAR vs. EP	31.00	− 1.53	0.165
	EC vs. HP	39.00	− 0.92	0.436
	EC vs. EP	29.00	− 1.75	0.123
	HP vs. EP	39.50	− 0.90	0.436

*Preset values, *p* < 0.05 in bold

*LAY* laypersons, *TAR* Traffic Accident Research staff, *EP* emergency physicians, *HP* hospital physicians, *EC* emergency services

#### Interdisciplinary differences after training

For the assessment of the front airbag (Table 3), no significant interdisciplinary difference dependent on training was observed in the Mann–Whitney *U* test (*p* ≥ 0.28).

## Discussion

In this study, a possible interobserver variability in the assessment quality of technical accident parameters was examined using real accident scenarios from GIDAS database collected by the Traffic Accident Research Institute at TU Dresden. The surveyed participants had varying degrees of experience in technical aspects and emergency medicine. Positive controls for technical understanding were employees of the TAR, while EP served as positive controls for preclinical emergency medicine knowledge. Laypersons with no prior experience in both fields served as the negative control group.

As the number of assessed cases increased, participants tended to require less time for parameter assessment, implicating a trainings effect. After user training, the processing time was significantly reduced (see Fig. 1). This suggests both a training effect and a positive impact of the training on processing time. After training, the median processing time for non-complex cases was 59 s.

A significantly positive training effect on the assessment quality was observed both in the overall analysis of all answers, regardless of parameters and professional group, and for the parameters rigid impact opponent, affected passenger compartment, EES, and front and side airbags. This indicates the necessity of training, especially for these parameters. These parameters were analyzed in a parallel project having significant impact on the prediction value of injury severity. Notably, both laypersons and medical professionals (doctors and emergency personnel) benefited from the training, indicating that prior medical knowledge played a relatively minor role. In contrast, TAR, who possessed technical knowledge, demonstrated superiority in the assessment, as expected. This was supported by their significant advantage in interdisciplinary comparison before training. For various parameters, such as passenger compartment damage, the superiority of TAR remained even after training, except for seatbelt usage (a preset value).

EC benefited significantly in the assessment of EES and damage to passenger compartment, possibly due to their frequent experience with accident scenarios in preclinical settings. This could suggest that EES and damage to the passenger compartment are of lesser concern and may have been easily defined through training. Vaca et al. postulate comparable results. The prediction of injury profiles by paramedics was sensitive, whereas the assessment of vehicle-specific crash variables was less accurate [3]. Doctors working in preclinical emergency medicine performed significantly better in three parameters, while clinic physicians and LAY performed better in four of the parameters assessed. This might be representative of their experience with traffic accident scenarios.

The training did not result in a significant improvement in the assessment quality for vehicle class, rollover, impact side, curtain airbag, or the predefined parameters of seat position, seatbelt usage, age, and gender. For vehicle class, it is plausible that distinguishing between compact, midsize, and luxury vehicles may not be straightforward, leading to minimal or even slightly worse assessments despite training. The other parameters mentioned already had high accuracy rates before training, so while training tended to improve them, it did not lead to significant improvements, especially for predefined parameters.

When examining complex scenarios, the significant advantages in assessing EES for EC, HP, and EP were no longer apparent. This confirms the expectation that in such scenarios, determining the main impact and its EES may be more challenging. Assessing the side airbag in complex collisions also appeared to be problematic, as it was no longer significantly better after training.

After training, significant interdisciplinary differences were only observed in the assessment of the front airbag, which were not confirmed in the Mann–Whitney *U* test. Accordingly, the training, which defined the parameters using image examples (not used in subsequent case scenarios following the training), led to a partial and significant improvement in the quality of assessment. On the other hand, the interdisciplinary differences before training were neutralized.

The results of our study reveal the practicability of preclinical assessment of injury severity predictive accident-related parameters. Following appropriate training, any person, regardless of medical or technical knowledge, can assess the accident parameters considered in this study, necessary for innovate injury severity prediction at the accident site. Without training, the assessment should primarily be conducted by individuals with technical expertise and emergency physicians. They performed best without training in assessing the rigid impact opponent, seatbelt usage, and airbag deployment. The EES should be trained for all professional groups if no prior knowledge is available.

Contrary to the seemingly problematic prehospital assessment of whether a severe injury is present [4–6], the technical parameters after a traffic accident appear to be well assessable through appropriate training.

In the absence of comparable studies, it is not possible to contextualize this work. However, it is known that training leads to improved performance of the participants [7, 8].

These facts offer the opportunity in advance the automatic eCall to a valid injury severity prediction tool allowing injury adapted alert of emergency medicine staff (HEMS, emergency physician, paramedics) and may help to decide which trauma center level is needed for the adequate treatment for the casualties.

With proper guidance from rescue center dispatchers or driver training, it would be conceivable to query these parameters in the context of an emergency call or eCall to make early inferences about potential accident outcomes and initiate appropriate primary alerting of rescue resources. A currently ongoing prospective multicenter study is investigating the feasibility and predictive accuracy of the aforementioned tablet-based injury prediction tool for prehospital optimization of care for trauma patients following traffic accidents. In this study, the mentioned parameters are being collected by various professional groups within the emergency medical services at both ground and air-based locations across multiple federal states to test the predictive validity. Results are pending. Regardless, this study provides robust results that after appropriate training, valid assessments of accident parameters can be made at the accident scene, regardless of professional background. For example, within a pre-project phase preceding the automated prediction tool (qualified eCall), dispatchers could query the necessary input parameters from the emergency medical service personnel present at the accident scene. This could be done using specific questionnaires to assess injury patterns and make appropriate decisions regarding the required rescue resources or disposition.

## Limitations

It is important to note that, in real accident scenarios, the views of the vehicles necessary for assessment may not be readily available, as they were during training. Therefore, a longer time commitment can be expected under real conditions, particularly in potentially stressful and chaotic accident scenes.

Furthermore, the statistical comparisons of individual collision directions are limited in their utility due to the low number of data points (two complex cases, three side impacts before and after training for each group). The parameters of seatbelt status, age, gender, and seating position were predefined in the survey, as they could not be determined from some accident images. Therefore, an analysis for these parameters is not possible.

The lower average professional experience of clinic physicians could potentially influence the comparability to the other professional groups, as they have less experience.

## Conclusions

In this study, we proofed the practicability of preclinical injury prediction based on relevant technical accident parameters. Significant differences in the quality of technical accident parameter assessment were observed depending on participants’ technical and medical backgrounds. Employees of the TAR were the most accurate in assessing parameters, while LAY, clinic physicians, EP, and emergency medical services (EC) personnel benefited from training in evaluating the specified parameters. Following the user training, the initial interdisciplinary differences were equalized, and all professional groups provided comparable assessment results without significant differences. It can be assumed that, after training or definition of these accident parameters using appropriate visual materials, any participant in the chain of care can make these assessments.

The time required for the complete assessment of the 14 accident parameters considered here decreased as the number of cases processed increased. Additionally, after training, the average time was significantly shorter than before, indicating a substantial learning effect through application and training. Thus, the collection of such parameters is trainable and can be done within a reasonable timeframe.

For future telemedical, preclinical queries of accident parameters, such as in the development of eCall or the establishment of injury prediction models, user training should be recommended to ensure an adequate assessment by users. After such training, the profession does not appear to have an impact on the quality of accident parameter assessment.

## Data Availability

No datasets were generated or analysed during the current study.
